# Nursing staff’s experiences of how weighted blankets influence resident’s in nursing homes expressions of health

**DOI:** 10.1080/17482631.2021.2009203

**Published:** 2021-12-14

**Authors:** Eva Hjort Telhede, Susann Arvidsson, Staffan Karlsson

**Affiliations:** a School of Health and Welfare, Halmstad University, Halmstad, Sweden; b Faculty of Health Science, Kristianstad University, Kristianstad, Sweden

**Keywords:** Experiences, health problem, nursing home, nursing staff, non-pharmacological intervention, older person, sleep problems, sleep-promoting, weighted blanket

## Abstract

**Purpose:**

The most common treatment for resident’s health problems is pharmacological. Little research has been done on how an intervention with a non-pharmacological method, such as a weighted blanket, Through the nursing staff view, we can learn how weighted blankets influence resident’s health in nursing homes. The aim of this study was to explore nursing staff’s experiences of how an intervention with weighted blankets influenced resident’s expressions of health.

**Methods:**

The study had a descriptive qualitative design with semi-structured interviews with 20 nursing staff working in nursing homes, and an inductive content analysis was applied.

**Results:**

The nursing staff expressed that the weighted blanket positively influenced resident’s health in the areas of sleep, physical activity, and psychological behaviour. The weighted blanket made them fall asleep faster, sleep was uninterrupted andthey felt more rested in the morning. The nursing staff observed an increased level of activity as the resident became more energetic . The nursing staff also experienced reduced negative psychological behaviours like anxiety and worrying.

**Conclusion:**

This study indicated that the weighted blanket changed the health expression of resident in several crucial areas. Deep pressure treatment indicates an alternative health-improved treatment for resident in nursing homes.

## Introduction

Older people’s health is influenced by several factors, not just diseases. As the proportion of older people in the world increases, the various health problems are also increasing. Although the increased number of older people is a very positive development, it means challenges. Older people’s health problems are both challenging and resource intensive, which leads to pressure on care providers’ services for older people (He et al., 2016). In previous studies, older people expressed that health experiences did not automatically have to do with diseases. On the contrary, many older people were aware that a certain degree of morbidity was a natural part of ageing. It was only when the diseases influenced the ability to experience independence that the perceived health of older people declined (Glasberg et al., 2014; Karppinen et al., 2016). Other important factors for the health of older people are to maintain mental and cognitive ability. Older people who have maintained cognitive ability with preserved personality and role function in life experience a higher degree of health (Glasberg et al., 2014; Karppinen et al., 2016). Social relationships and participating in physical and mental activities also positively influence health in older people (Glasberg et al., 2014; Hörder et al., 2013; Malderen et al., 2016; Sini et al., 2015; Sundsli et al., 2013). Supporting older people ‘s activity, independence, and cognitive capacity can positively influence their health regardless of their underlying physical or mental illness.

Health problems in older people with physical and mental illness can lead to problems such as sleep problems and loss of appetite. In addition, these aggregate dilemmas often strengthen each other negatively, which worsens the health problems (Gulia & Kumar, 2018). It also makes the health problems of older people complex because the circumstances of the health problems influence and reinforce each other. These health problems become apparent when older people move in as residents of nursing homes, leading to increased challenges in maintaining health and independence. (Overbeek et al., 2018). In addition to physical illnesses that many resident experiences in the nursing home, it is also usual to have health problems such as depressive symptoms and anxiety problems. One in three women and almost one in five men in the oldest age group (85 years or older) suffer from anxiety and depressive symptoms (Gonçalves et al., 2011; Lennartsson & Heimerson, 2012). Both anxiety and depressive health problems are correlated with sleep disturbances to varying degrees, indicating the complexity of the health of resident as they often have numerous ill-health factors influencing each other (Gulia & Kumar, 2018; Overbeek et al., 2018). Chronic stress has also been identified as an essential factor in many older people’s sleep problems. The complexity increases because older people who experience anxiety and stress have an increased risk of experiencing mental illness (Braam et al., 2014; Segel-Karpas et al., 2017). Sleep problems can occur as a result of anxiety, depression, and stress, but sleep problems can also lead to anxiety, depression, and stress. These health problems are also affected by social factors (such as loneliness or hospitalization), which become more common in the older population (MacLeod et al., 2018; Sagayadevan et al., 2017). The opportunity for older people to experience health is a multifaceted experience, and health in older people involves more than just treating diseases. Numerous circumstances reinforce each other, and sleep problems are one of these factors that negatively influence health.

Inadequate sleep affects the health of older people regardless of the underlying circumstances behind the lack of sleep. Insufficient sleep also means a higher risk of falls and other accidents. Poor sleep also intensifies and contributes to the progression of dementia (Brewster et al., 2015; Phelan et al., 2010; Sagayadevan et al., 2017; Shi et al., 2018; Wennberg et al., 2013). The health problems connected with inadequate sleep, whether they are secondary or primary, also influence older people’s psychosocial and social capabilities with more limited enthusiasm for social interaction. Sleep problems can also be associated with increased agitation (Ryden et al., 2019; Suzuki et al., 2017; Webster et al., 2020). In nursing homes, it is common for the resident’s health problems to be treated pharmacologically. Pharmacological treatment (e.g., hypnotics, sedatives, antidepressants) is today a first-line treatment strategy in nursing homes as a sleep-promoting and anxiety-suppressing treatment, although these drugs are not recommended for resident due to an increased risk of severe side effects (Bloom et al., 2009; Hill et al., 2007; O`Neill et al., 2020; Pitkala et al., 2015; Salzmann-Erikson et al., 2016; Zuidema et al., 2015). These unwelcome side effects of pharmacological treatments may lead to other interventions in the care of resident (Bloom et al., 2009; Hellström et al., 2014).

Alternative non-pharmacological interventions can attenuate expressions associated with unhealthy conditions, which can occur with increasing age. The unhealthy conditions can mean mild or severe cognitive impairment, insomnia and mental illness, most often pharmacologically treated (Voyer et al., 2005). There are side effects of pharmacological treatment that can influence resident’s health in many ways. The nursing staff works close to the resident and are important because they experience changes in the resident’ s health.

When older people end up in some form of care as residents, they must adapt to a considerable extent to the new situation. They become dependent on nursing staff and therefore lose a large part of their control over their lives. The nursing staff’s responsibility becomes essential in everyday life for residents. Much of the daily routines is governed by the nursing staff’s routines and their ability to understand the needs and expressions of residents (Jarling et al., 2018). When there is a reduction in resident’s self-determined ability, nursing staff need to make sense of everyday life and assess the resident’s needs. When residents and the nursing staff understand each other, it is experienced as satisfying. The nursing staff who work narrowly with the residents can also experience the consequences of health-related problems and view variations. Therefore, the experiences of nursing staff are essential in assessing and understanding the influence on health expression that the non-pharmacological implementation as a weighted blanket may have on residents (Ancoli-Israel, 2009; MacLeod et al., 2018; Slettebø et al., 2017; Webster et al., 2020). The non-pharmacological intervention of a weighted blanket creates deep pressure on the body and dampens the sympathetic response associated with, for example, anxiety and sleep problems (Mullen et al., 2008; Reynolds et al., 2015).

Theories behind deep pressure therapy have their origins in the theory of sensory integration, which describes how different sensory stimuli affect how the brain processes sensory information. Deep pressure is a part of the sensory integration theory and leads to reduced anxiety and worry (Kimball et al., 2007; Reynolds et al., 2015). Methods that provide deep pressure, such as weighted blankets, have been reported to have valuable influences by reducing several conditions that unfavourably influence health. The effect of deep pressure is described as calming, providing improved sleep, reducing anxiety, and generally increasing well-being. However, most studies performed with this form of intervention have been performed among populations with psychiatric and neuropsychiatric diagnoses and not among older people (Morrison, 2007; Sylvia et al., 2014). Thus, there is a lack of research on non-pharmacological methods such as a weighted blanket to improve sleep and health in resident living in a nursing home. As the population of older people increase, the incidence of health problems will increase, making assessing and treating this problem essential. Due to systematic use of weighted blankets for resident in nursing homes with sleep problems, we could understand through the nursing staff’s experiences the influence that a weighted blanket has on health expression as an alternative solution to the resident’s health problems in nursing homes, especially the high incidence of sleep disorders.

## 2 Aim

The aim was to explore nursing staff’s experiences in how an intervention with weighted blanket influenced resident’s in nursing homes expression of health.

## 3 Methods

### 3.1 Design

The study had a descriptive qualitative design with semi structured interviews with nursing staff in nursing homes and followed an inductive content analysis (Graneheim & Lundman, 2004).

### 3.2 Intervention

Written and oral information was given to the nursing staff at the introduction of the weighted blanket. The weighted blanket was first tested when the residents were in a normal sleeping position, with the soft side against the person’s body, starting at the feet. The nursing staff were urged not to leave the residents alone but to stay and follow their reaction, to observe that residents could remove the weighted blanket independently. The chains in the weighted blanket were not designed to prevent residents from moving. If there were no side effects, the weighted blanket was raised farther up the body. The nursing staff was instructed not to place the weighted blanket twice over the chest and not to place the blanket too tightly around the resident’s bodies. The nursing staff was encouraged to remove the weighted blanket if the residents showed signs of discomfort. If they expressed that the weighted blanket felt too heavy, but they still wanted to try it, the nursing staff was told to contact the researchers to get a weighted blanket of lighter weight. In the absence of effect, the nursing staff was encouraged to contact the researchers to change to a heavier weighted blanket. Information on how the weighted blanket test was to be performed was also provided in writing to all nursing staff. The weighted blanket used in the study was made of chains and weighed about 10% of each participant’s body weight A weighted blanket with about 10% of a person’s weight has been shown to have a calming effect (Mullen et al., 2008). In this study, the weighted blanket varied between 4 and 8 kg, depending on the weight of the resident. The chains in the weighted blankets were sewn in channels, and the fabric was durable and fireproof. Hygiene covers were not used due to the risk of suffocation. Most of the resident used the 6 kg weighted blanket, and those who slept with the weighted blanket were 65 years or older with sleep problems and were living at a nursing home. Sleep problems for these residents was defined based on Diagnosis of Insomnia (ICD 10- SE) (The National Board of Health and Welfare, 2010). The study period of 28 days was based on previous studies where the influence on sleep by the weighted blanket was shown after 2–4 weeks (Ackerley et al., 2015; Hvolby & Bilenberg, 2011). If the older person was cold with the weighted blanket, an ordinary blanket was also placed over the older person and then the weighted blanket. Resident in palliative care or who had severe muscle loss, severe lung disease, or heart failure were excluded.

### 3.3 Sample

The study included 23 participants who worked as nursing staff in seven comparable nursing homes in municipalities in the southwest of Sweden. Three of the participants rejected the interview the day before the interview—two stated that their high workload was the reason, and the third stated illness as the reason. Those who dropped out of the study came from different nursing homes. Twenty participants were included in an individual interview, and 90% had an (nursing) education at the secondary school level. The term nursing staff used in this study refers to nursing staff who provide care in nursing homes for resident. They had the main responsibility for the everyday care of the residents in the nursing homes, for example, helping with meals, dressing, washing, and toileting. The participants ranged in age from 25 to 70 years, and professional experience as nursing staff varied between 4 months and 45 years. A purposive sampling was done among participants who had the best knowledge concerning the research topic of how an intervention with a weighted blanket might influence resident’s expression of health. The participants were identified and recruited by the manager of each nursing home. The nursing home managers selected the participants who worked night shifts or were contact persons for an older person who had used a weighted blanket for 28 days. A preliminary request for participation was distributed together with written information about the aim of the study and the consequences of being included. Interested participants left their name and email address with the nursing home manager, who in turn provided the information to the researcher. All participants who received information via email from the researcher agreed to participate in the interview.

### 3.4 Data collection

Data collection with interviews was provided between February 2020 and December 2020. The ten-month time delay occurred due to a ban on visiting nursing homes in connection with Covid-19. The researcher and the participants planned a meeting for an interview during the participants’ working hours. Before the interview, the researcher made sure that the participant had received written information about the study and understood what it meant to participate, that the study was voluntary, and that they could drop out at any time without consequences. At the physical meeting prior the interview, a written informed consent was obtained from the participant. An interview guide with semi-structured open-ended questions was used. The main questions in the interviews were: *Can you describe what older person’s sleep was like before the introduction of the weight blanket, Tell me if you have experienced any health changes in resident who used weight blankets at your nursing homes*, and *Describe the older person’s activity during the day after using the weighted blanket*. Two interviews were conducted as pilot interviews. After the pilot interviews, four more questions were added to the interview guide. The pilot interviews were included in the study. The interviews lasted 25–60 minutes. All interviews were conducted in premises near the workplace and were recorded digitally and then transcribed.

### 3.5 Data analysis

The interviews were analysed with qualitative content analysis (Graneheim & Lundman, 2004). The transcribed text was read several times, and the transcribed interviews were discussed in the research group. The text was then divided into meaning units and abbreviated into condensed meaning units. The condensed meaning units were abstracted and labelled with a code. The different codes were sorted based on similarities and differences and sorted into subcategories. A process of reflection and discussion resulted in an agreement on how the codes should be sorted. The codes were revised, reviewed, and discussed in the research group and then condensed into categories (Graneheim & Lundman, 2004). Examples of meaning units, condensed meaning units, codes, subcategories, and categories are shown in Table I.

**Table I.** Example of the analysis process

Meaning units	Condensed meaning units	Codes	Subcategories	Category
She was already asleep when we arrived. However, before that, the alarms came immediately (7)	Asleep when we arrived. Before, the alarm came immediately	Falls asleep early Fewer alarms	Falls asleep faster Undisturbed night’s sleep	Sleep quality

## 4 Results

Three categories were derived from the nursing staff’s interviews regarding their experiences of resident’s health expressions after using a weighted blanket. The categories were *Influenced sleep*, which meant that the nursing staff experienced that the resident improved their sleep by falling asleep more quickly and were sleeping more undisturbed at night, *Influenced activity*, which meant that the older person had more energy to participate in physical activities, more energy to have conversations, and more energy to eat independently, and *Influenced psychological behaviour*, which meant that the residents showed calmer expressions and behaviours where they could express their desires in a more nuanced way with less anxious outbursts. The residents who did not like the weighted blanket expressed it clearly and early during the intervention.

## 5 Influenced sleep

The nursing staff experienced that the weighted blanket influenced the sleep of the residents in many ways. This influenced sleep meant that nursing staff experienced that the residents fell asleep faster in the evening, got an uninterrupted night’s sleep, and slept longer in the morning. The residents who were usually awake were already sleeping with the weighted blanket in the evening when the nursing staff began their night shift. The residents who were awake when the nursing staff started to work often fell asleep immediately when they got the weighted blanket. The nursing staff described that they could observe how the resident’s body relaxed and sank into a state of calmness with the weighted blankets. The relaxation became most evident in the residents who were mobile in bed before the weighted blanket was introduced. The mobility of the residents in the bed meant that they got up and down from the bed and twisted and turned incessantly without sleeping.

*We got her ready, put on the weighted blanket, and we could see over her whole body that she had relaxed and was in a cosy atmosphere* (Interview 8).

The nursing staff also experienced that many residents began to express the desire to go to bed and that this desire came in connection with introducing the weighted blanket. The change to more consistently improved sleep also became apparent when the room alarm decreased at night. It was not uncommon for the residents to use the room alarm long after going to bed at night and then repeatedly at night. In some cases, the residents completely stopped triggering the alarm in the room during the night when using the weighted blanket, and in other cases partially.

*She rang the alarm pretty much all the time, and we were coming and then she did not know what she wanted once we were there, and she was very confused during these periods before the weighted blanket* (Interview 14).

Before the weighted blanket was introduced, it was common for the residents to wake up several times during the night and express the need to go to the toilet. Several of the nightly toilet visits yielded no results. The nursing staff experienced that it was as if the residents did not know what they wanted when they woke up at night but interpreted it as a need to go to the toilet. When the residents started sleeping with the weighted blanket, the proportion of toilet visits decreased.

*There is a man here who sleeps much better with his weighted blanket. He thinks it is cosy when you put it on him. He is not up that much anymore. He woke up more in the past, it was like a toilet fixation, really. It is not certain that there is a need for the toilet, and it is more of just “I have woken up, then I have to go to the toilet”* (Interview 19).

The residents who slept with the weighted blanket were not as tired and did not wake up as early as before the weighted blanket was implemented. The nursing staff described that the number of sleeping pills was reduced as the sleep improved. Giving the residents a smaller number of sleeping pills was something that the nursing staff experienced as very positive. The nursing staff experienced that sleeping pills increase the risk of falls and that sleeping pills do not always lead to the residents falling asleep or sleeping all night and could lead to more wakefulness during the night.

*This, with the sleep of the residents, has gotten so much better with the weighted blanket[…]With medicines there is a risk of falling, and other injuries are possible. I do not know if the medicines do any good. A lot of the medicines have probably been left standing, I think, since a long time ago* (Interview 12).

The nursing staff also experienced that the weighted blanket had been a link to better sleep. Several residents continued to maintain good sleep after the study period with the weighted blanket. When it came to resident with dementia, the nursing staff had different experiences. Some nursing staff claimed that the weighted blanket did not help with sleep problems in the residents with advanced dementia. Other nursing staff experienced that the weighted blanket worked very well and improved resident’s sleep even if they had a dementia disease.

*Of course, they have dementia, but I still think I have imagined that the blanket had a good effect on them* (Interview 12).

The weighted blanket did not work for all residents, and those who did not like the weighted blanket showed it clearly and early in the introduction of the weighted blanket, mainly connected to bedtime. They expressed dissatisfaction by throwing the weighted blanket on the floor or by telling or showing discomfort with a facial expression. Although the resident did not express discomfort from the weighted blanket, the nursing staff described that the same sleep problems could persist despite the weighted blanket. The nursing staff felt that the residents who did not like closeness and hugs had a greater challenge in accepting the weighted blanket. The nursing staff also described how their own behaviour could have an impact on whether the residents liked the weighted blanket or not. If the nursing staff was stressed at bedtime, the residents had a more challenging time accepting the weighted blanket. The nursing staff experienced that a calm approach from the nursing staff at bedtime led to increased peace and higher acceptance by the residents for the weighted blanket. The nursing staff experienced that there may be other reasons for sleep problems that must be understood when the weighted blanket did not improve sleep. The discomfort was mainly seen in the residents who usually did not sleep with a regular blanket. The nursing staff expressed that those who did not usually sleep with a regular blanket mainly expressed their dissatisfaction with the weighed blanket, and this was something that the nursing staff said and to be considered in the implementation.

*He got agitated immediately when he got the weighted blanket on. It turned out that he did not usually sleep with a regular blanket, so of course he did not want a weighted blanket, and he wondered what we were doing to him* (Interview 3).

### 5.1 Influenced activity

The nursing staff experienced that the resident’s activity was influenced when they started with the weighted blanket, and the change occurred about 3 weeks after implementation. The nursing staff experienced that the residents turned back the clock, which meant that they were more awake during the day and could participate in daily activities. They also became more active in social contexts, showed more independence, and were more active in meal situations.

*She was tired all the time before, so there we see a difference, she has got a different routine […] More order in everything that is good for the body* (Interview 5).

The nursing staff described that the morning routine became more manageable after the implementation with the weighted blanket. It was easier to get the residents motivated to start the day, less persuasion was needed, and the residents expressed energy for the activities. The nursing staff experienced that the increased energy level was especially noticeable during the morning when the residents got up, and it became easier to get the residents out of bed. Before the weighted blanket, many residents sat and slept during the day without participating in any activities. The residents who previously only sat and slept during the day partially reduced their sleep daytime when they started using the weighted blanket, while others stopped sleeping entirely during the daytime. The residents who got better naps during the day became more active during the day. This change was related to using a weighted blanket. Before the weighted blanket, the residents lay in bed almost all day, slept in a chair or could sit and snooze all day without any distinction between activity and rest. The experience of the nursing staff was that more balance was created between rest and activity. The changed activity level also led to the residents sitting up longer and being more active in the evening.

*She became more involved in activities. Yes, she sat more in the wheelchair before. After about a month or three weeks, we could get her up more and then she could sit and listen to music […] and then we could take away the wheelchair […] then she could just walk, and we just thought, ‘Oh my god’* (Interview 8).

The nursing staff experienced that many residents who previously did not want to be alone changed after using the weighted blanket, and they performed several activities more independently. Before the weighted blanket, many residents just wanted to sit in their wheelchair during the day with no energy for independent activities. As their energy increased, activities became more independent. Independence was shown by the residents walking and moving around by themselves without any wheelchair or other aids on more occasions than before. The nursing staff also experienced that the residents independently wanted to make their beds themselves. However, they described the weighted blanket as heavy and challenging to handle, something that the nursing staff found frustrating for the residents.

*He makes the bed himself, and it may happen that the weighted blanket fell off. It is pretty heavy for him. He tried to fix it himself and so on, but at the same time, he slept much better. So it’s a bit like that (laughs*) (Interview 1).

Activity also increased in terms of the energy to participate in conversations. The increased energy for participating in conversations was shown by the residents giving increased feedback and by being more active in asking follow-up questions. Previously, they had not shown the same activity in the conversations, and for the most part they just sat quietly without interest in the conversation. The increased communication was directed to the nursing staff and other residents in the nursing homes.

*When you talked to her, she answered, and it was in the right context, and maybe even a question came back so you could sit and have a conversation. She managed to have a conversation, quite simply* (Interview 2).

The residents who used the weighted blanket also became more socially active with other residents in the nursing homes. The increased social activity made the residents more interested in their surroundings and increased their involvement in more activities. Before implementing the weighted blanket, many residents avoided social contexts and activities. The interest in participating in the social community and physical activities gradually increased during the intervention. The activity around mealtimes changed, where the ability to feed oneself increased. Some residents needed continued support in meal situations, while others could handle their meal situation more independently. The nursing staff also described how many residents before the weighted blanket could not chew their food or use utensils. Previously the residents might fall asleep in the middle of the meal. The meal-time activity changed when the residents could chew their food, use utensils, and drink from a glass. The nursing staff experienced that the proportion of residents who could eat on their own increased as the level of activity increased.

*Before we had to feed her in the morning because she was so tired, but now she can eat her sandwich by herself* (Interview 2).

### 5.2 Influenced psychological behaviour

The nursing staff experienced that the consequence of implementing the weighted blanket influenced the resident’s ability to formulate their feelings more clearly. As a result of this change with more peaceful emotional and psychological expression, less pharmacological treatment was needed during the day. The more relaxed psychological-emotional expressions that residents showed meant less anxiety and worry and less aggressive behaviour. Behaviours such as anxiety, worry, and aggression were psychological expressions for which the residents previously received pharmacological treatment. In some cases, pharmacological treatment was required, mainly with benzodiazepines, but not to the same extent when the weighted blanket was used. The decreased pharmacological treatment was applied to both on-demand drugs and standing prescriptions.

*When she got the weighted blanket, we removed some medicines because she was not as worried anymore, and she began taking fewer on-demand drugs because she was not just as worried anymore, so it was very good. I experienced that her whole demeanour had changed* (Interview 8).

The nursing staff expressed that before the weighted blanket, it was not uncommon for the residents to express disorderly and anxious behaviours, which made it challenging for nursing staff to understand what the residents wanted. This confusing behaviour gradually changed, and the nursing staff experienced an increased understanding between the residents and the nursing staff when the residents manifested calmer psychological behaviour.

*Before the weighted blanket, they became messy, and they became aggressive as well, so they did not understand things, so it did not connect, it was almost like a psychosis […] now they are very kind and happier as well, so they have completely different personalities (*Interview 9).

The nursing staff experienced changes in the resident’s cognitive abilities in connection with the introduction of the weighted blanket, and the residents showed an increased degree of psychological and cognitive understanding of the nursing staff’s communication. The nursing staff felt that it was previously a challenge to understand the residents when emotional expressions manifested through different psychological behaviours. The behaviour could be expressed by the residents biting on nearby things such as furniture, making inexplicable picking movements, or screaming. Psychological behaviour of this kind calmed down after the implementation of the weighted blanket. The nursing staff described how they experienced that some of the residents had undergone a personality change since the weighted blanket was used.

*It was very upsetting behaviour, and before the weighted blanket he wanted to tear things and was very verbally unpleasant. So, it is clear that we are noticing a big personality change with the weighted blanket* (Interview 7).

With the weighted blanket, the resident’s psychological expressions became easier to handle, and the emotional expressions became easier to understand, according to the nursing staff. The residents also complained less when they became psychologically calmer, and the previously anxious, stressed, aggressive, irritated, and frustrated emotional expressions were replaced by more psychologically cooperative behaviours and a higher degree of patience.

*She is no longer as angry and annoyed. She is actually in a better mood. Before the weighted blanket, she complained about everything, and nothing was good, and she felt so bad, and it was such a shame for her. Now there is not as much of it anymore. She is much calmer* (Interview 19).

The nursing staff experienced that the residents had a change in psychological and emotional behaviour by becoming more active in the conversations by showing a higher ability to express different psychological dimensions. The residents showed emotions such as joy through more laughter than before, and they became more aware of their surroundings. The residents gained a greater understanding of the discussions through changed psychological behaviour in social contexts. With the changing psychological behaviour, the residents became more inclined to communicate and became more socially close to others in the nursing homes. The residents consistently showed their emotions through calmer and clearer psychological behaviours as their cognitive ability increased.

*You can even get such an excellent comment like, “I want to sit with you and eat”. It is so good with fellowship thinking when you are alert and happy, and it gives you self-confidence instead of hiding in your room. That is nice* (Interview 5).

The weighted blanket was also used when the residents were sitting in a wheelchair or another chair during the daytime. The weighted blanket could then be placed over the resident’s legs and arms. Using the weighted blanket during the day influenced the residents to become less contact-seeking. The contact-seeking behaviour was previously manifested by the residents constantly yelling at the nursing staff without formulating what they needed.

*We have an old man, he asks for the weighted blanket during the day and then he calms down, he does not scream as much […] I know I asked him about the weighted blanket, and he has experienced it very, very positively in that it hugged him. He says it held him!* (Interview 20).

The nursing staff expressed how the resident’s behaviour was influenced by more psychologically positive expressions than negative expressions. The nursing staff also described that the longing for food was expressed more than before the weighted blanket, where the interest in food was previously non-existent. Even expressions such as that the food tasted better increased when the residents were longing for the food.

*So before she got the weighted blanket, she could lie down all day and did not want to get up and so on. We wanted to see if a weighted blanket helps, and after three weeks we got her up, and she could be social and laugh and talk and thought the food tasted good, and just, ‘Oh this was good, I have never eaten this’, we just, how good* (Interview 8).

## 6 Discussion

The use of weighted blankets provides various improvements in sleep among the residents in nursing homes. The nursing staff experienced that healthy sleep was attained because the weighted blanket helped the residents fall asleep faster and to sleep more undisturbed through the night. Similar influences of the weighted blanket have been reported from previous studies (Ackerley et al., 2015; Bundy & Lane, 2020; Ekholm et al., 2020; Reynolds et al., 2015). Previous studies were not performed on the older population per se, but on more mixed target groups. Adjusting sleep with weighted blankets has been described as a tactile non-pharmacological complement to improve sleep quality (Ackerley et al., 2015). In this study, the nursing staff experienced a reduction in pharmacological treatment and that this reduction was related to implementing the weighted blanket. The weighted blanket as a complementary health-promoting treatment can be considered as an innovation that can improve sleep or supplement the pharmacological practice in nursing homes. In addition to the point that pharmacological treatment increases the risk of side effects in the residents, they also have other interacting health-influencing factors such as multi-morbidity and ageing itself that affect opportunities for good sleep (Gulia & Kumar, 2018). This phenomenon of possible reduced pharmacological treatment in connection with the implementation of weighted blankets is interesting in clinical practice but needs to be studied more. In this study the improvement in sleep occurred relatively quickly. This rapid influence from weighted blankets was also observed in a previous study by measuring the vital parameters related to the use of a weighted blanket. The reaction occurred after only five minutes, with notable changes in decreased blood pressure and heart rate and increased calm (Reynolds et al., 2015). There are advantages to the fact that the weighted blanket’s influence shows up quickly during implementation. This facilitates measuring the effect of the weighted blanket because it is possible to experience the result of the intervention quickly. This study is unique because it was performed on the residents in nursing homes. Studies with a weighted blanket are normally conducted on a more mixed target group, usually young people.

The consequences of the weighted blankets are often described as a result of the deep pressure the weighted blanket exerts. This influence with a focus on deep pressure treatment has often been described in young people with different sensory integration dysfunction conditions (SPD). The dysfunction includes disorders with an inability to interpret sensory signals leading to anxiety, motor control problems, behavioural problems, and depression (SPD Foundation, 2016). Attention has been drawn to the point that deep pressure also shows sleep improvements in individuals other than those with SPD (Ackerley et al., 2015) The improvement in sleep in the residents may be illustrated by the influence of deep pressure on the central nervous system (CNS) (Bundy & Lane, 2020; Reynolds et al., 2015). Sensory responses in the nervous system are often interpreted as a reflection of the autonomic nervous system, and when someone acts defensively or stressfully there is an overreaction in the sympathetic component of the autonomic nervous system (Bundy & Lane, 2020). Interestingly, this attenuation in the sympathetic part of the CNS might also can explain why the residents utilized the weighted blanket in the current study even if they did not have known SPD problems. However, more studies are needed to understand the relationship between deep pressure treatment with weighted blankets on fragile residents. To our knowledge, only one study has been performed on the target group of residents, and the weighted blanket was used on older persons with dementia for five months. The results from their study were similar to the results from our study, where the consequences of using the weighted blanket were, among other things, generally improved sleep (Nakamura & Yamauchi, 2021). More clinical trials on the target group of residents in nursing homes and more participants are needed to support these results. Previous studies highlight the prevalence of sleep problems in resident and the importance of maintaining and improving healthy sleep in old age (Brewster et al., 2015: Siddarth et al., 2020). Morrison (2007) highlighted that studies performed with deep pressure treatment using weighted vests on children with inattention and hyperactivity problems exposed methodological weaknesses in ensuring that the treatment with weighted vests worked adequately (Morrison, 2007). Indeed, they used deep pressure treatment with weighted vests, while our study used weighted blankets, which can have different influences on sleep. Regardless of this, our results are compatible with previous research suggesting that sleep is improved using the weighted blanket (Ackerley et al., 2015; Ekholm et al., 2020: Nakamura & Yamauchi, 2021). It is essential to emphasize that the residents in this study who did not like the weighted blanket showed it early in the implementation.

Interventions with weighted blankets may lead to increased activity in the daytime in the residents in nursing homes. The observations of the changed activity level came gradually after the residents slept with the weighted blanket. One explanation for this could be that when the residents get better sleep, their ability and strength to perform activities increase. Previous research has found a link between reduced sleep time and reduced ability to engage in physical activity. Previous studies show that decreased sleep time with several awakenings during the night is associated with low physical activity during the day (Medic et al., 2017; Song et al., 2015). In our study, the nursing staff experienced that the improved activity level influenced the residents in several activity areas, and this was accompanied by increased independence in everyday activities. Thus, many indications are that the improved level of activity has positive consequences for resident’s quality of life and health experience. Improved activity and independence are prerequisites for healthy ageing regardless of illness, as highlighted in previous studies. Higher activity levels give the vitality that increases the opportunity to experience health (Karppinen et al., 2016; Medic et al., 2017; Ryden et al., 2019; Suzuki et al., 2017) Previous studies emphasize that insomnia in older persons can contribute to the lack of energy that prevents coping with daily activities (Ryden et al., 2019; Suzuki et al., 2017). The link between sleep deprivation and reduced activity could be understood as circumstances that negatively influence the health (Medic et al., 2017) The results of previous studies are entirely in line with the experiences that nursing staff presented in our study. The changes in the health of residents give the weighted blanket a vital position for health improvement, which cannot be ignored. Further study is needed to determine whether interventions to improve sleep will delay the decline in functional activity in the residents living in a nursing home.

Weighted blankets have a positive influence on the psychological behaviours of the residents living in a nursing home. Observations from the nursing staff were that the residents showed calmer expressions and behaviours and could express their wishes in a more nuanced way. A generally calmer behaviour with less anxious outbursts could be attributed to the deep pressure effect of the weighted blanket and the known outcomes of deep pressure treatment (Bundy & Lane, 2020). Stimulation of deep pressure induces changes in autonomic arousal and suppresses outward behaviour and expression (Reynolds et al., 2015). The current study showed that resident had a greater ability to understand their surroundings, which indicates that fewer misunderstandings arose between the nursing staff and the residents. This result is meaningful and indicates that improved cognitive ability in the residents can lead them to be more able to make themselves understood. Previous studies showed that maintaining cognitive ability is vital for the health of the residents because they associate high cognitive ability with the experience of feeling healthy (Karppinen et al., 2016; Medic et al., 2017). The current study showed that when the residents have a mentally calmer behaviour, they create conditions to participate in social contexts and express themselves in a more nuanced manner. Essential components for the health of the residents can be the possibility for autonomy and meaning in social contexts. This is highlighted in previous studies where meaningfulness and experiences of autonomy are essential for the experience of healthy ageing (Karpinnen et al., 201 2016). Cognitive impairment and accelerated brain ageing are effects of long-term sleep deprivation (Brewster et al., 2015; Carvalho et al., 2017; Shi et al., 2018) In addition, accelerated brain ageing associated with sleep deprivation can lead to the development of Alzheimer’s disease and vascular dementia (Carvalho et al., 2017; Shi et al., 2018). The improvements that occurred in the residents in the present study are easier to understand based on long-term sleep deprivation on cognitive ability. The expressions of psychologically calmer behaviour and increased ability to express their desires in a more nuanced way became easier to follow. The negative psychological behaviours during the day that occur in connection with sleep deprivation are significantly associated with increasing memory problems, a frequent but underestimated symptom (Siddarth et al., 202 2020) It is apparent that adequate and high-quality sleep promotes cognitive psychological health expression and memory problems (Dzierzewski et al., 2018). Moreover, these negative psychological expressions and behaviours can be easy to miss in fragile residents with comorbidities. To our knowledge, this is the first study investigating whether a weighted blanket can influence resident’s health expressions in nursing homes. By using nursing staff to describe the influence of the weighted blanket on the resident’s health, we had the opportunity to gain access to several different health results. Several of the health expressions can be partly understood based on the theory of deep pressure and sensory integration because the weighted blanket reduces autonomous arousal. This connection is not completely clear in the previous study and needs to be studied further (Bundy & Lane, 2020). Just measuring the nursing staff’s observations says nothing about how the residents experienced the weighted blanket. Objectively measuring the sleep influence confirmed the outcome of the result on sleep, although more studies with larger sample sizes are needed in these cases. In summary, our results suggest that the weighted blanket has a good influence on resident’s expressions of health in nursing homes.

## 7 Strengths and limitations

To maintain confirmability, the researchers strived to be neutral and objective throughout the research process (Malterud, 2014). The researchers’ pre-understanding could affect the research process at all levels. However, pre-understanding can also be advantageous in this study by creating the research question, which facilitated the researchers understanding of the nursing homes’ problems and conditions. Nevertheless, the pre-understanding has also contributed to and justified the research that was carried out. It was important for the researchers to raise awareness of pre-understanding through discussions between the researchers in all phases of the study. By conducting discussions about pre-understanding, it became easier for researchers to keep the pre-understanding in the background through all the research process steps, which made it easier at all stages of the research to reproduce the participants’ words (Malterud,) 2014. When the results were presented, the participants’ own words were reflections of what they experienced happened to the residents, not in their own experiences, but their described experiences. Therefore, the study was performed with manifest content analysis with an inductive method. To further confirm the participants’ own words, several quotes from the various interviews were presented. In this way, neutrality can be maintained, and the distance between the researchers and those participating in the study were clarified (Elo et al., 2014; Polit & Beck, 2018). The interviews were semi-structured, which meant that the standardized questions allowed follow-up questions, which gave more in-depth answers as the participants had the opportunity to develop their answers. The interview guide was created in advance and tested through two pilot interviews. After the pilot interviews, questions were added to clarify and receive a more accurate description of the investigated phenomenon. When nothing new emerged in the interviews, a few more interviews were conducted to ensure that data saturation had occurred (Elo et al., 2014; Polit & Beck, 2018) It is common for residents of nursing homes to have cognitive disabilities in the form of dementia. Nevertheless, despite the cognitive barriers, residents’ health must be preserved (Chua et al., 2016). In this study, the nursing staff as proxy observed health differences even if residents were not able to respond for themselves. In previous studies, the nursing staff has acted as a proxy for the residents. Proxy ratings can provide a unique insight into residents’ health and can be a contributing factor in improving the health in cognitively impaired residents (Robertson. et al., 2019)

Credibility was strengthened by conducting the data collection with 20 interviews at seven different nursing homes in the county. The selection was made strategically with the nursing staff who worked close to the residents who used weighted blankets. The interview included six nursing staff who worked at night caring for at least one older person who used the weighted blanket. The 14 nursing staff who worked during the day were asked when they were the contact persons for the residents who used weighted blankets. The advantage of using nursing staff who were contact persons was that they knew the habits and behaviours of the residents well. The nursing staff who worked at night met several residents who used the weighted blanket, which meant that they could see the resident’s changes. The prerequisite for the credibility of the study increased because the nursing staff knew the residents well (Malterud, 2011; Polit & Beck, 2018). The nursing staff were of different ages and with varying work experiences. A weakness in the study was that fewer men were included, which might mean that the results might have been different if more men participated. However, the gender distribution reflects what it looks like in nursing homes, making it difficult to influence the study’s selection method to obtain an equal gender distribution in the sample. The results were also presented with quotes from many different interviews, which increased the credibility of the results (Polit & Beck, 2018).

When it comes to the transferability of the results, this is contextual. The similarities in the interviews mean that it is possible to transfer the results in similar contexts. However, it is a weakness that it is difficult to know whether the results from this study can be transferred to other contexts outside nursing homes (Polit & Beck, 2018). Throughout the research process, the researchers have compared their thoughts based on individual assessments of everything from raw data in the interviews to the coding process and the creation of categories.

During the compilation of meaning units, condensed meaning units, codes, subcategories, and categories, the researchers regularly analysed the material independently of each other (Graneheim & Lundman, 2004). The results were discussed, and similarities and differences between the researchers were compared. Adjustments to the material were made after several discussions until an agreement was reached between the researchers. Discussions, comparisons of suggestions, and adjustments in the material were made during all the parts of the study. Categories were abstracted in discussions between the researchers until consensus was reached. Throughout the analysis process, the researchers regularly returned to the raw data material to see that they had stayed close to the interviews (Polit & Beck, 2018). This study’s presentation of the results increases the authenticity as the study also reports a lack of influence on some of the residents’ health concerning the weighted blanket. By reporting the participants’ different experiences, the researchers reported a representative and varied picture in order to increase the opportunities to relate to the different perspective’s authenticity. Data stability over time involved the authors conducting interviews over a long time and at several different nursing homes, with questions constructed through agreement among the authors. The interviews took place in an environment under conditions in which the participants felt comfortable in speaking openly about their opinion as confidently as possible (Polit & Beck, 2018).

## 8 Conclusion

This study indicated that the use of weighted blankets changed the health expression of the residents in several crucial areas. It is challenging to determine which health changes occurred initially, but it can be assumed that they influenced each other. The nursing staff viewed that sleep improved, anxiety was reduced, anxious outbursts decreased, and energy and the ability to participate in activities during the daytime increased among the residents. All in all, the weighted blanket created health-promoting expressions, which could partly be explained by the theory of sensory integration in which deep pressure calms the overactivity of the sympathetic nervous system. Deep pressure treatment with weighted blankets might be an alternative health-promoting treatment for the residents. However, more studies are needed to explore the influence of weighted blankets on the residents in nursing homes.
